# Satisfaction and Perceived Coercion in Voluntary Hospitalisations: Impact of Past Coercive Experiences

**DOI:** 10.1007/s11126-022-10005-8

**Published:** 2022-10-24

**Authors:** Debora Martinez, Alexandra Brodard, Benedetta Silva, Oana Diringer, Charles Bonsack, Stéphane Morandi, Philippe Golay

**Affiliations:** 1 Community Psychiatry Service, Department of Psychiatry, Consultations de Chauderon, Lausanne University Hospital and University of Lausanne, Place Chauderon 18, 1003 Lausanne, Switzerland; 2 Institute of Psychology, Faculty of Social and Political Science, University of Lausanne, Lausanne, Switzerland; 3 Cantonal Medical Office, Directorate General for Health of Canton of Vaud, Department of Health and Social Action (DSAS), Avenue des Casernes 2, 1014 Lausanne, Switzerland; 4 Psychiatric Center of Neuchâtel (CNP), 2074 Marin-Epagnier, Switzerland; 5 General Psychiatry Service, Department of Psychiatry, Lausanne University Hospital and University of Lausanne, Lausanne, Switzerland

**Keywords:** Perceived coercion, Satisfaction, Involuntary hospitalisation, Long-term impact, Perceived humiliation

## Abstract

Coercion in psychiatry is associated with several detrimental effects, including in the long term. The effect of past experience of coercion on the perception of subsequent hospitalisations remains less studied. The present study aimed to assess the impact of past experience of coercion on the perception of coercion and satisfaction with subsequent voluntary hospitalisations. A total of 140 patients who were hospitalised on a voluntary basis were included. Fifty-three patients experienced coercion before this hospitalisation and 87 did not. Patients were assessed for treatment satisfaction and perceived coercion. Health status was also evaluated by both patients and carers. Past experience of coercion was the independent variable. Perceived coercion and satisfaction scores were used as different dependent variables in a series of regression models. Results suggested a long-term detrimental impact of past experience of coercion on some aspects of satisfaction and perceived coercion in subsequent voluntary hospitalisations even when controlling for self and carers-rated health status. Overall, this study suggests that special attention should be paid to patients who are voluntarily admitted to hospital but have a history of coercion, as they may still be impacted by their past coercive experiences. Ways to increase satisfaction and reduce perceived coercion of these patients are discussed.

## Introduction

The use of coercion in mental health care remains a sensitive topic since it infringes the bioethical principal of patient’s autonomy and its efficiency is still discussed in the field [1, 2]. In practice, caregivers have the duty to protect patient’s health and safety as well as other people against the danger patient may represent [3]. Despite the will to improve psychiatric treatment and make it more respectful, it is very difficult to completely avoid coercion. Thus, caregivers are struggling between the two bioethical principles of beneficence and autonomy [4].

Patients generally perceive coercion negatively and many studies have highlighted the multiple harmful effects of perceived coercion on them [5, 6]. Formal coercion is a measure imposed against patient's will, without their agreement or knowledge, such as confinement, prohibition of free movement or contact with relatives, seclusion, restrain and forced medication. It differs from informal coercion in the sense that this is based on an official decision. Informal coercion consists of persuasion, interpersonal pressure, inducements, threats and warnings, carried out by careers or relatives without a formal legal decision [3]. Formal coercion has shown a negative impact on patients’ quality of life [7] but also on their clinical course. Indeed, formal coercion reduces satisfaction with care [8] and treatment adherence in the long-term [9]. Perceived coercion (i.e. the feeling of being coerced) increases disengagement from services and negative therapeutic relationships [10–12]. This negative impact on health care process can adversely affect patient’s prognosis due to potentially difficult cooperation [6]. Experience of coercion can also be highly stressful [13]. Moreover, according to Jordan and McNeil [14], higher level of perceived coercion during the psychiatric hospital’s admission process increases the risk of suicide attempts after release, potentially endangering the patient’s life. Several studies have also showed the strong impact of the feeling of being coerced on satisfaction with care [15–17]. Finally, past coercion (i. e. a previous experience of formal coercion) is also linked to a higher risk of use of formal or informal coercion in the future [18, 19], potentially leading patients into a vicious circle of poor therapeutic collaboration and the deterioration of their situation.

Evidence about the long-term effect of perceived coercion remains scarce but few studies suggested a worsening of the patients’ attitudes towards treatment and mental health care, which constitutes a barrier to the use of psychiatric services [20]. Past involuntary hospitalisation is related to an increased sensitivity over time to the feeling of being coerced to treatment, leading to lower treatment adherence levels [18, 21]. In their two-year follow-up study, Van der Post et al. [21] added support to the hypothesis that there may be an association between patients’ perception of their past compulsive admission and the risk of re-hospitalisation.

Overall, current studies suggest negative effects of coercive measures on patients, although there is no clear evidence of a causal relationship between past coercion and the perception of future readmission. This is an understudied effect and several authors recommended further studies in order to better understand the complex association between perceived coercion and care [22–24].

In order to contribute on this matter, the aim of this study was to assess the impact of past formal coercion experiences on 1) perceived coercion and 2) on satisfaction during voluntary hospitalisation. For methodological reasons, past coercion was defined for the patients as at least one formal experience of confinement, prohibition of free movement or contact with relatives, seclusion, restrain and forced medication. Our goal was to examine how perception of coercion and satisfaction in voluntary hospitalisation can be accounted for by the impact of past coercion experiences. We chose to focus on current voluntary hospitalisation so that patients were in the same context and the only variable that could affect patients' perceptions was the presence of a past experience of coercion, after controlling for self- and carer-rated health status. We hypothesized that long-term detrimental impact of past experience of coercion on satisfaction and perceived coercion could be highlighted in subsequent voluntary hospitalisations.

## Material and Methods

### Participants

Participants were all hospitalized and were recruited throughout six psychiatric hospitals in the French-speaking part of Switzerland between March 2020 and June 2022. The hospitals were covering several districts in three cantons (Vaud, Neuchâtel, Fribourg) and comprised acute psychiatric wards. Inclusion criteria were to be between 18 and 65 years old, being hospitalised from 7 and 15 days and sufficiently proficient in French to complete the questionnaire. This ensured participants formed a rather homogeneous group in terms of duration of hospitalisation elapsed at the time of assessment. Patients diagnosed with dementia (F00-F09) or intellectual developmental disorders (F70-F79) were excluded. Patients were contacted directly on site to take part in the study on a voluntary basis by trained research assistants. In order to avoid selection bias and to approach more often those patients who are known to be cooperative, each hospital unit drew up a list of eligible patients, who were then selected at random. Approval for this study was granted by the Human Research Ethics Committee of the Canton Vaud, Switzerland (protocol #2016–00,768). Written informed consent was obtained from all participants and all methods were carried out in accordance with the recommendations of the Human Research Ethics Committee of the Canton Vaud and the Declaration of Helsinki.

### Measures

#### Socio-demographic Data

Socio-demographic characteristics such as age, gender, nationality, occurrence of previous psychiatric hospitalisations and main diagnosis were collected through structured questionnaires and medical charts. Past experience of coercion was defined as past involuntary hospitalisation, confinement, prohibition of free movement or contact with relatives, isolation, physical or drug restraint.

#### The MacArthur Admission Experience Survey (AES) Short Form

The French version of the MacArthur Admission Experience Survey (AES) short form was used to measure patients’ perceived coercion at admission [5]. This 16-item dichotomous (true or false) scale is divided into three subscales and a total score. The Perceived Coercion score focuses on freedom, choice, initiative, control and influence over coming into hospital; the Negative Pressures score focuses on being forced, threatened or physically forced to come into hospital; and the Voice score focuses on having a chance to voice an opinion about coming into hospital. The final item consists of a range of adjectives used to assess the patient’s affective reaction to hospitalisation. Although easy to use and very short, the AES does not cover all aspects of coercion and only refers to the hospital admission process [25].

#### The Coercion Experience Scale (CES)

The French version of Coercion Experience Scale [CES; 26] was used to measure patients’ perceived coercion during their hospitalisation. The scale consists of 31 items, the first 2 items being 0 to 100 visual analogue scales to assess the extent to which patients remember the coercive measures (item 1) and the extent to which these were considered stressful (item 2). The second part of the questionnaire is based on 29 five-point Likert-type items divided into five subscales: a Humiliation/coercion score, a Physical adverse effects score, an Interpersonal separation score, a Negative environmental influences score and a Fear score.

#### Satisfaction Regarding Hospitalisation (ANQ)

The Swiss National Association for Quality Development in Hospitals and Clinics (ANQ) has developed a satisfaction measure for patients in psychiatry. The questionnaire includes 6 five-point Likert-type items assessing quality of treatment, information and communication, medication, patient’s implication and discharge preparation [27]. A total score can be computed to assess the global satisfaction of the patient.

#### Health of the Nation Outcome Scales (HoNOS-F)

The French version of the Health of the Nation Outcome Scales (HoNOS-F) is a routine clinical tool used in order to assess the health and social functioning of people with mental illness [28]. In Switzerland, the HoNOS is routinely used and rated by clinicians for every hospital admission and discharge. The measure has originally been developed in the UK between 1992 and1995 and has since been translated and validated in different languages. In this study, we used the French version translated and validated by Lauzon et al. [29] which it is composed of twelve 5-point Likert-scale items. Several scoring structures have been proposed for the HoNOS scales, some of which were deemed unsatisfactory for the French version. Therefore, as recommended in a large sample study on the French version [28], scores were selected at the item-level and all twelve scores were used.

#### Self-reported Health

One subscale of the ANQ satisfaction regarding hospitalisation short questionnaire is a self-reported five-point Likert-type item about the patient self-perceived global health.

### Statistical Analysis

In order to estimate the relationship between past coercive experiences and satisfaction and perceived coercion during voluntary hospitalisation, we used a series of linear multiple regressions. Past experience of coercion was introduced as the independent variable. All HoNOS items and self-reported health were also introduced in order to control for patients’ health. Age, gender, Swiss nationality, occurrence of previous psychiatric hospitalisation and diagnostic were also controlled for. Coercion and satisfaction scores were used as different dependent variables. All statistical analyses were performed using IBM SPSS Statistics 27.

## Results

A total of 140 voluntarily admitted psychiatric patients took part in this study. 53 patients (37.9%) experienced coercion before this hospitalisation and 77 patients (55.0%) were women. Age ranged from 18 to 63 years old (*M* = 39.26, *SD* = 14.15). All patients were French-speakers, and the large majority (75.0%; *n* = 105) was Swiss. Primary diagnosis, based on the *International Statistical Classification of Diseases and Related Health Problems 10*^*th*^* Revision* (ICD-10) are presented in Table 1. 71.4% (n = 100) reported at least one previous psychiatric hospitalisation.

**Table 1:** Sample description (N = 140)

Gender, female, % (n)	55.0 (77)
Age, mean (sd)	39.26 (14.15)
Swiss Nationality, % (n)	75.0 (105)
Past Experience of Coercion	37.9 (53)
Previous Psychiatric Hospitalisation, % (n)	71.4 (100)
Main Diagnosis, % (n)	
Mental and behavioural disorders due to psychoactive substance use (F10)	7.9 (11)
Mental and behavioural disorders due to psychoactive substance use (F11-F19)	2.9 (4)
Schizophrenia (F20-F29)	15.7 (22)
Mood affective disorders (F30-F31)	10.7 (15)
Mood affective disorders (F32-F39)	31.4 (44)
Neurotic, stress-related and somatoform disorders (F40-F48)	10.0 (14)
Personality disorders (F60-F69)	17.1 (24)
Psychological development disorders (F80-F89)	0.7 (1)
No diagnostic information available	3.6 (5)

Relationships between past experience of coercion, perceived coercion and satisfaction scores were estimated (Table 2). The AES perceived negative pressures score (β = 0.272, p = 0.016) was significantly predicted by past experience of coercion. The CES perceived fear score (β = 0.220, p = 0.037) was also significantly predicted by past experience of coercion).

**Table 2:** Relationship between past experience of coercion and perceived coercion/satisfaction

Effect of past experience of coercion on outcomes	B	*β*	*p*-value
Outcomes			
Perceived coercion at admission (AES)			
Perceived Coercion subscale	0.377	.116	.312
Negative Pressures subscale	0.862	.272	.016*
Voice subscale	0.057	.033	.760
Total score	1.385	.203	.073
Perceived coercion during hospitalisation (CES)			
Humiliation/Coercion subscale	5.289	.178	.072
Physical Adverse Effects subscale	0.410	.091	.406
Interpersonal Separation subscale	0.259	.056	.585
Negative Environmental Influences subscale	0.768	.109	.312
Fear subscale	0.732	.220	.037*
Satisfaction regarding hospitalisation (ANQ)			
Quality of Treatment	-0.356	-.158	.113
Asking Questions	-0.442	-.219	.036*
Getting Answers	-0.293	-.120	.280
Medication	-0.657	-.208	.053
Implication	0.256	.091	.362
Discharge	0.054	.019	.865
Total score	-1.544	-.132	.177

All models were adjusted for HoNOS items, self-reported health status, age, gender, swiss nationality, previous psychiatric hospitalisation and diagnostic

*p < .05

One aspect of self-reported satisfaction with treatment (ANQ) was also negatively related to past experience of coercion: *asking questions* (β = -0.219, p = 0.036). Relationship between dependent variables and covariates are presented in Table 3.

**Table 3:** Correlations between dependent variables and covariates

	1	2	3	4	5	6	7	8	9	10	11
1. Past coercion experience	-										
2. AES Perceived Coercion	0.12	-									
3. AES Negative Pressures	.18^*^	.64^*^	-								
4. AES Voice	-0.05	-.48*	-.31^*^	-							
5. AES Total score	0.16	.90^*^	.85^*^	-.64^*^	-						
6. CES Humiliation/Coercion	.20^*^	.31^*^	.36^*^	-.35^*^	.40^*^	-					
7. CES Physical Adverse Effects	0.16	0.07	0.02	-0.02	0.04	.41^*^	-				
8. CES Interpersonal Separation	0.08	0.13	0.07	-.19^*^	0.13	.66^*^	.35^*^	-			
9. CES Negative Environmental Influences	0.11	0.06	0.13	-0.07	0.1	.47^*^	.38^*^	.38^*^	-		
10. CES Fear	.25^*^	0.03	0.03	0.01	0.02	.21^*^	.26^*^	0.11	0.17	-	
11. ANQ Quality of Treatment	-0.08	-0.1	-0.13	0.14	-0.14	-.50^*^	-0.13	-.52^*^	-.25^*^	-0.07	-
12. ANQ Asking Questions	-0.16	-0.09	-0.15	0.08	-0.12	-.36^*^	-0.17	-.50^*^	-.23^*^	-0.12	.57^*^
13. ANQ Getting Answers	-0.15	-0.12	-0.14	0.13	-0.14	-.52^*^	-.25^*^	-.49^*^	-.29^*^	-0.16	.54^*^
14. ANQ Medication	-0.15	-0.14	-.20^*^	.23^*^	-.21^*^	-.39^*^	-0.11	-.31^*^	-0.14	-0.05	.50^*^
15. ANQ Implication	0.06	-.20^*^	-.25^*^	0.15	-.25^*^	-.48^*^	-.24^*^	-.45^*^	-.17^*^	-0.08	.52^*^
16. ANQ Discharge	0	-0.03	-0.05	.20^*^	-0.09	-.46^*^	-0.04	-.44^*^	-0.13	-.20^*^	.48^*^
17. ANQ Total score	-0.1	-0.15	-.21^*^	.21^*^	-.21^*^	-.60^*^	-.21^*^	-.59^*^	-.26^*^	-0.14	.77^*^
18. Honos 1	0.16	0.02	0.09	-0.06	0.06	.22^*^	.22^*^	0.13	.24^*^	0.15	0.12
19. Honos 2	-0.09	0.04	-0.02	0.06	-0.01	0.05	0.09	0.01	-0.09	0	-0.1
20. Honos 3	-0.11	-.20^*^	-0.08	0.09	-0.16	0.09	0.01	0.15	0.01	-0.13	-0.08
21. Honos 4	0.08	0.06	0.03	0.04	0.03	-0.13	0.1	-0.09	-0.03	0.06	.20^*^
22. Honos 5	-0.06	0.13	-0.02	-.23^*^	0.11	0.14	0.16	.21^*^	0.02	-0.09	0.01
23. Honos 6	.30^*^	-0.1	-0.12	0.15	-0.14	0.01	0.09	-0.04	-0.09	.34^*^	0.13
24. Honos 7	-.21*	0.06	0.09	.18^*^	0.03	-0.01	0.04	0.05	-0.1	-0.04	-0.1
25. Honos 8	-0.13	0.06	0.06	0.03	0.04	0.07	.183^*^	0.05	0.05	.18^*^	-0.02
26. Honos 9	-0.03	0.04	0.08	-0.02	0.06	0.09	0.14	0.12	.18^*^	.22^*^	-0.09
27. Honos 10	-0.01	-0.04	0.01	-0.04	-0.01	0.03	0.1	0.15	0.12	0.01	-0.09
28. Honos 11	-0.03	0	0.05	-0.06	0.04	-0.1	0.01	-0.05	-0.11	0.02	0.1
29. Honos 12	-0.05	-0.05	0.02	0.01	-0.02	0.14	.19^*^	0.13	0.08	0.05	-0.09
30. Age	-0.03	0.07	0.08	-0.09	0.08	.17^*^	0.06	0.13	0.13	0.15	-0.15
31. Female gender	-.22*	-0.01	0.1	-0.14	0.07	-0.1	-0.06	0.1	-0.01	-0.02	-0.03
32. Swiss nationality	0.14	.18^*^	0.09	-0.02	0.13	.17^*^	0.14	0.07	.20^*^	0.15	-0.13
33. Previous hospitalisation	.46^*^	0.03	0.07	-0.01	0.05	0.02	0.16	0.09	0.08	0.07	-0.04

	12	13	14	15	16	17	18	19	20	21	22
1. Past coercion experience											
2. AES Perceived Coercion											
3. AES Negative Pressures											
4. AES Voice											
5. AES Total score											
6. CES Humiliation/Coercion											
7. CES Physical Adverse Effects											
8. CES Interpersonal Separation											
9. CES Negative Environmental Influences											
10. CES Fear											
11. ANQ Quality of Treatment											
12. ANQ Asking Questions	-										
13. ANQ Getting Answers	.50^*^	-									
14. ANQ Medication	.47^*^	.62^*^	-								
15. ANQ Implication	.45^*^	.53^*^	.55^*^	-							
16. ANQ Discharge	.40^*^	.45^*^	.30^*^	.53^*^	-						
17. ANQ Total score	.71^*^	.79^*^	.78^*^	.80^*^	.69^*^	-					
18. Honos 1	-0.01	-0.06	-0.07	-0.15	-0.03	-0.06	-				
19. Honos 2	-0.12	0	-0.03	0	-0.03	-0.04	-0.08	-			
20. Honos 3	0	-0.02	0.05	-0.02	-0.01	-0.01	0.04	-0.1	-		
21. Honos 4	0.05	0.08	0.02	0.02	0.15	0.11	0.09	-0.07	-0.02	-	
22. Honos 5	-.18^*^	-0.1	0	-0.04	-0.04	-0.07	0.01	-0.07	-0.04	.24^*^	-
23. Honos 6	0.07	-0.04	0.04	0.08	0.14	0.09	0.16	0.05	-0.16	.30^*^	-0.07
24. Honos 7	-0.14	0.06	0.09	-0.06	-0.08	-0.03	-.18^*^	.34^*^	0.01	-0.01	0.07
25. Honos 8	-0.05	-0.02	-0.08	-.21^*^	-0.02	-0.1	0.13	0.14	-0.02	.17^*^	0
26. Honos 9	-0.14	-0.12	-0.01	-0.08	-0.04	-0.09	0.09	0.04	-0.01	0.14	0.09
27. Honos 10	-.23^*^	-0.11	-0.14	-0.15	-0.01	-0.16	0.06	-0.08	-0.11	.41^*^	.23^*^
28. Honos 11	-0.14	0.08	0.15	-0.02	-0.07	0.03	0.09	0.06	-0.07	0.15	0.12
29. Honos 12	-0.17	-0.12	-0.1	-0.1	-0.07	-0.12	0.1	0.12	.17^*^	.21^*^	.17^*^
30. Age	-.23^*^	-0.05	-.23^*^	-.25^*^	-.18^*^	-.24^*^	-0.01	0.14	-.17^*^	-0.04	0.04
31. Female gender	-0.04	0.03	-0.01	-.17^*^	-0.08	-0.06	0.02	-.23^*^	0.07	.18^*^	.20^*^
32. Swiss nationality	-0.04	-0.1	-0.07	-0.15	-0.14	-0.15	0.03	0.04	-0.16	-0.02	0
33. Previous hospitalisation	-0.03	-0.07	0	-0.06	0.02	-0.05	0.11	-.28^*^	0.12	0.15	0.01

	23	24	25	26	27	28	29	30	31	32	33
1. Past coercion experience											
2. AES Perceived Coercion											
3. AES Negative Pressures											
4. AES Voice											
5. AES Total score											
6. CES Humiliation/Coercion											
7. CES Physical Adverse Effects											
8. CES Interpersonal Separation											
9. CES Negative Environmental Influences											
10. CES Fear											
11. ANQ Quality of Treatment											
12. ANQ Asking Questions											
13. ANQ Getting Answers											
14. ANQ Medication											
15. ANQ Implication											
16. ANQ Discharge											
17. ANQ Total score											
18. Honos 1											
19. Honos 2											
20. Honos 3											
21. Honos 4											
22. Honos 5											
23. Honos 6	-										
24. Honos 7	-.19^*^	-									
25. Honos 8	0.08	.29*	-								
26. Honos 9	0.05	0.06	0.16	-							
27. Honos 10	0.13	0.11	0.08	.35^*^	-						
28. Honos 11	-0.01	.23^*^	0.07	0.16	.42^*^	-					
29. Honos 12	0.09	.18^*^	0.11	.30^*^	.34^*^	.24^*^	-				
30. Age	0	0.06	.213^*^	0	0.05	0.09	-0.04	-			
31. Female gender	-.23^*^	-0.01	-0.05	0.01	.19^*^	0.15	0.08	0.03	-		
32. Swiss nationality	0.13	-0.14	-0.03	0.13	0.03	0.04	-0.14	0.07	-0.09	-	
33. Previous hospitalisation	0.14	-.24^*^	-0.07	0.06	0.11	-0.02	0.06	-0.13	0.12	0.07	-

* p < .05

## Discussion

Our study examined the relationship between past coercion and future voluntary hospitalisations. Our results suggested a significant detrimental association between past coercion experiences and perceived negative pressure and fear as well as treatment satisfaction for being able to ask questions during hospitalisation after controlling for patients’ self-rated and carer-rated level of health and functioning.

Our findings suggested that patients with a history of coercion are more likely to perceive negative pressure and fear during re-admission and hospitalisation and also be less satisfied with treatments and information, i.e., the ability to ask questions. This is important because it suggests that past coercion negatively affects patients’ subsequent hospitalisation even when these are voluntary. Studies have shown that past coercion can be highly stressful [30, 31]. The relationship between past coercion experience and fear highlight the difference between “objective” and “perceived” coercion and reminds the concept of “coercive shadow”: a patient’s perception of threat, even where no threat may be intended, and the fear of patients that non-compliance may lead to the use of coercion. Patients therefore commonly agree to treatment, including “voluntary” hospital admission to avoid the humiliation and stigma of a compulsory order [32]. Without going that far, past experiences of coercion certainly play a role in the patient's perception of psychiatric care during subsequent voluntary hospitalisations. It also has been shown that fear of treatment can be detrimental to therapeutic alliances and impact health [33]. There are several clinical implications: voluntary patients with a past coercion experience are less likely to adhere to treatment and more likely to have a negative experience of voluntary hospitalisations. Thus, special consideration should be given to patients who have a history of coercion in order to mitigate this potential negative impact from the past.

Regarding the reduction of the feeling of coercion, recent work has identified procedural justice i.e., the feeling of being treated with respect and receiving a fair decision, as an important mean [10, 20]. Silva et al. [34] findings also support this conclusion showing that perceived fairness plays an important positive role in satisfaction and is therefore a key ingredient to improve care.

Additionally, as suggested by the work on patient-centred care, which advocates for care respectful of individual patient preferences, values, and needs, active patient participation in the process is essential to improve satisfaction with care [10, 15, 20, 35, 36]. Within this framework, shared decision-making is the primary means to promote a balanced patient-physician relationship that distances itself from the patriarchal model and promotes changes that address the needs identified in the present study such as voice and access to understandable and quality information [35]. Enhancing the use of shared decision making is therefore a crucial matter to work on in order to increase patients’ satisfaction.

These two propositions are intrinsically linked since the feeling of fairness stems from the feeling of being considered, notably through involvement in care as proposed by the shared decision-making model. In line with the goals of patient-centred care, tools such as the Joint Crisis Plan [JPC; 37] are available to put these propositions into practice. Other possibilities for future research need to be explored, such as identifying modifiable variables that are associated with satisfaction and perception of coercion during voluntary hospitalisations for patients with a history of coercion. We could for example explore the choice of location (distance from home and relatives, contact with other patients, painful history related to location) or the life trajectory (hospitalisation at a turning point in life).

Our study has some limitations. First, our sample size was modest and further research should replicate our results in larger samples. Second, despite our attempt to reduce the selection bias by approaching randomly selected patients, we cannot exclude that the decision to participate or not may have had an impact on our results. Indeed, some patients with a rather negative treatment experience might have avoided participation in our study or, on the contrary, dissatisfied patients might have felt a great need to share their negative experience. Third, we did not have a complete inventory of past coercion experiences or past hospitalisation for each patient and relied on a self-rated indication. Being rather coarse (yes/no), we were not able to estimate whether a dose–response effect of past coercion could be highlighted. This matter should be further investigated in future studies.

## Conclusions

Overall, this study indicated that patients with a history of coercion are less satisfied with some aspects of care and are more likely to perceive negative pressures and fear in subsequent voluntary admissions and hospitalisations. This relationship between past coercion and satisfaction was evident even after controlling for patient’s self- and carers-rated level of health and functioning. Therefore, special attention should be paid to patients who are voluntarily admitted to hospital but have a history of coercion as they may still be impacted by their past coercive experiences. This study highlights the necessity to further develop patient-centred care in order to increase long-term satisfaction with psychiatric care.
